# Influence of External Light on Ultra-Weak Photon Emission of Fruits: Forensic Differentiation of Organic and Conventional Fruits

**DOI:** 10.3390/s25061799

**Published:** 2025-03-14

**Authors:** Adrián Rubio, Anna Barbaro, Gemma Montalvo, Fernando E. Ortega-Ojeda, Carmen García-Ruiz

**Affiliations:** 1 Departamento de Química Analítica, Química Física e Ingeniería Química, Universidad de Alcalá, Ctra. Madrid-Barcelona, km 33.6, 28871 Alcalá de Henares, Madrid, Spain; adrian.rubio@uah.es (A.R.); a.barbaro@uah.es (A.B.); gemma.montalvo@uah.es (G.M.); fernando.ortega@uah.es (F.E.O.-O.); 2 Instituto Universitario de Investigación en Ciencias Policiales, Universidad de Alcalá, Calle Libreros, 27, 28801 Alcalá de Henares, Madrid, Spain; 3 Studio Indagini Mediche E Forensi (SIMEF), Via Nicolò da Reggio 4, 89128 Reggio Calabria, Italy; 4 Departamento de Ciencias de la Computación, Universidad de Alcalá, Ctra. Madrid-Barcelona, km 33.6, 28871 Alcalá de Henares, Madrid, Spain

**Keywords:** ultra-weak photon emission (UPE), light exposure, spectral imaging, fruit colour, organic produce, food fraud detection

## Abstract

Ultra-weak photon emission (UPE) provides a non-invasive method for assessing the biochemical state of biological materials. In this work, we investigated UPE in fruits of various colours (red, orange, yellow, and green) for potential forensic applications. Firstly, fruits were exposed to natural sunlight for 10 min, after which UPE was measured at 10 min intervals over a three-hour period. The results indicated that, following the initial induced response, all fruit types stabilised to a spontaneous UPE state after approximately 60 min in darkness. Subsequently, we compared UPE responses following exposure to natural sunlight with those obtained under artificial red, green, and blue lights. Under natural sunlight, induced UPE values ranged from 15 to 35 intensity units (IU) and spontaneous UPE from 1 to 25 IU, whereas under artificial lighting, induced UPE ranged from 5 to 30 IU and spontaneous UPE from 1 to 20 IU. Finally, a preliminary comparative study between organic and conventional fruits revealed that organic fruits consistently emitted slightly higher UPE levels than conventional ones, suggesting subtle differences in their biochemical properties. All these findings underscore the potential of UPE as a forensic tool for differentiating plant-based materials, with promising applications in food fraud detection and criminal investigations.

## 1. Introduction

The forensic analysis of plant-based materials has traditionally focused on certain key species; however, fruits have remained underexplored despite their potential forensic value in criminal studies [1,2]. For this reason, the evaluation of fruits needs to be more significant in forensic research, due to their relevance in the investigation of various types of felonious acts, ranging from determining the time of death in cases where fruits are found at the crime scene [3,4,5] to analysing food fraud [6,7]. Hence, fruits play a significant role in forensic science for multiple reasons. They can be key evidence in understanding the environmental conditions surrounding a crime scene [8,9,10,11,12,13], assessing the time elapsed since some fruit was exposed to specific conditions [14,15], or identifying food-related incidents [16,17]. As a result, forensic scientists often need to evaluate the freshness and ripeness of fruits involved in environmental and food fraud cases. Accurate determination of these factors can be crucial in reconstructing events and fully understanding the evidence [18].

It has been demonstrated that the evaluation of the state and physical properties of fruits can provide critical information in important criminal contexts [19,20]. For these studies, forensic analysis often relies on well-known traditional methods for assessing fruit ripeness and quality, such as visual inspection, which relies on observable changes in colour and texture; firmness tests, which measure the mechanical resistance of the fruit; and chemical assays, which can quantify specific compounds associated with ripeness or spoilage. While effective, these methods can be invasive or limited in scope. However, advancements in technology are presenting new opportunities for gathering valuable information by non-destructive techniques. One such advancement could be the implementation of ultra-weak photon emission (UPE) measurement as a novel tool for fruit analysis [21].

Fruits undergo a series of biochemical changes as they ripen, affecting their texture, colour and chemical composition. These changes are driven by various physiological processes, including the production of ethylene, a hormone that regulates ripening, and the increase in reactive oxygen species (ROS) during maturing, which are the main cause of UPE [22]. Understanding these processes is essential in forensic chemistry, as they influence the fruit’s appearance and chemical properties.

UPE is a phenomenon where biological systems emit extremely low levels of light, undetectable to the human naked eye. This emission is linked to the presence of ROS and other metabolic by-products produced during biochemical processes. The characteristics and intensity of UPE can reflect the physiological and biochemical state of the organisms, including fruits. UPE has been primarily attributed to the relaxation of electronically excited ROS formed during oxidative processes, such as lipid peroxidation and the oxidation of proteins and nucleic acids [23]. These excited species undergo an electronic transition to the ground state, emitting photons, mainly in the visible (VIS) light range [24,25,26]. Consequently, measuring UPE in biological samples offers a unique perspective for numerous scientific applications, including forensics.

The use of UPE in fruits aligns with its established application in other biological systems, as it has been utilised to study oxidative stress and cellular metabolism in plants and humans. Its ability to reveal subtle biochemical changes makes it a promising tool for enhancing the forensic analysis of fruits. However, despite its promising applications, UPE remains largely unexplored in fruit research, with only a few studies addressing its potential [27,28,29,30,31,32,33,34]. More notably, no prior research has investigated its forensic applications, and this gap serves as motivation for our study, which aims to provide an innovative approach to fruit analysis by employing biophoton emission measurements.

Among the few existing investigations involving UPE in fruits, some recent studies have explored its application in food quality [27], for example, analysing maturity and ripeness in tomatoes [28,29] and strawberries [30]. Consequently, it is showed how this technique could offer a unique advantage by providing non-invasive insights into the fruit’s internal condition [31], such as assessing germination levels in watermelon seeds [32], the medicinal properties of traditional Chinese herbs [33] and the impact of a pulse electric field on strawberries, raspberries and blackberries [34]. By analysing their UPE, researchers can assess ripeness, detect early signs of spoilage and evaluate the effects of different storage conditions without damaging the fruit. Nevertheless, these studies have primarily focused on using UPE as a tool for monitoring ripening or food quality, leaving a critical gap in its forensic application for food fraud detection (organic vs. conventional produce).

Furthermore, exposure to external light is recognised in the literature as one of the most influential factors affecting UPE [35]. Thus, irradiation of a living organism with direct light causes its external surfaces (e.g., skin or peel) to absorb photons, subsequently increasing its UPE by various orders of magnitude over a short period of time until stabilisation is achieved. This “induced” UPE phenomenon is known as delayed luminescence or delayed light emission (DLE). Accordingly, the study of both induced and spontaneous UPE following external light exposure is particularly relevant for analysing the internal characteristics of fruits, which is a key factor in detecting food fraud. Notably, existing studies on fruits rarely compare the effects of different light sources on both induced and spontaneous UPE, nor do they address the kinetics of UPE stabilisation after light exposure.

As a result, this work aims to evaluate both the induced and spontaneous UPE variations in fruits of varied colours (red, orange, yellow and green) as a function of both the variety and ripeness stage when exposed to different light sources, including artificial red, green and blue (RGB) lights and natural sunlight, in order to (i) analyse the temporal evolution of biophoton emission from fruits to determine the optimal dark adaptation period for obtaining reliable spontaneous UPE; (ii) compare the effects of natural sunlight and artificial RGB lights on both induced and spontaneous UPE and (iii) explore the potential of UPE to differentiate between organic and conventional fruits.

## 2. Materials and Methods

### 2.1. Samples

This study was conducted using 16 different samples, selected for their varied colours (red, orange, yellow and green), as representative of the most common fruits. They were organised in three sets of samples (A, B and C) to measure them according to our custom sampler (2 × 3 box):

Set A. This set included three different colour variations (red, green and yellow) of two types of fruits: apple (*Malus domestica*) and pepper (*Capsicum annuum*).

Set B. This set comprised the same three colour variations of other fruit types: plums (*Prunus domestica*) and grapes (*Vitis vinifera*).

Set C. This set consisted of four types of citrus fruits: an orange (*Citrus × sinensis*) and a tangerine (*Citrus reticulata*), representing orange fruits; a lemon (*Citrus × limon*), representing yellow fruits; and a lime (*Citrus × aurantifolia*), representing green fruits.

Although UPE measurements can be applied to a wide range of fruits and plant-derived materials, our study focused on a specific selection to investigate the influence of colour variations and cultivation practices. As a result, the first two sets of fruits were intentionally chosen to include different colour variations within the same species and maturity degree, thereby allowing us to assess the potential effects of fruit colour on UPE. In addition, a set of citrus fruits was included to further evaluate the influence of colour on UPE, even when fruits belong to different species but within the same genus. Finally, complementary samples were selected to analyse the potential differentiation between organic and conventional fruits; in this case, red and green peppers and red apples from both cultivation types were examined.

### 2.2. Instrumentation

**Figure 1:**
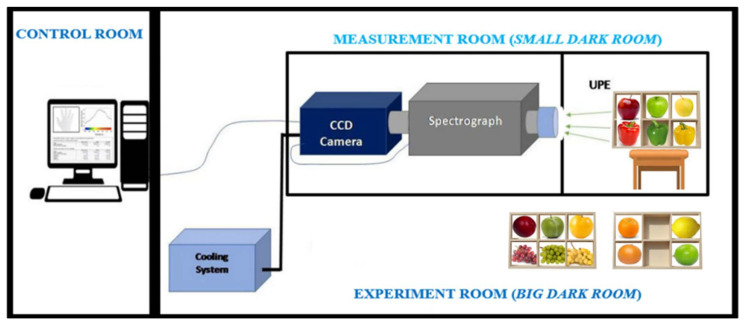
Structure of the rooms used for the UPE measurements in fruits, showing the arrangement of the three sets of fruits in a six-compartment sampling box (image not to scale).

For this study, we used an updated version of a custom-built system originally designed in one of our previous studies to measure human UPE [36]. Briefly, the structure of this system consisted of two contiguous rooms: one containing the control and acquisition system and the other housing the device setup, as shown in Figure 1.

The experimental and device setup was specially designed for measuring UPE images of six fruits simultaneously. For this purpose, this design included one custom sampler (2 × 3 box) for placing the fruits at a distance of 2 m from the measuring instruments.

The system also included a fixed aperture Nikon f/1.2 28 mm lens (Nikon Corporation, Tokyo, Japan) connected to the entrance of a spectrograph (Kymera 193i-A, Andor, Belfast, UK), in turn, connected to an almost-zero-dark-noise, high-sensitivity charge-coupled device (CCD) camera (iKON M-934, with maximum quantum efficiency in the 400–800 nm range, Andor, Belfast, UK). The intercalated spectrograph was only used in mirror mode to direct the light to the camera.

This system and the fruits were placed inside a measurement room specifically designed to isolate them from any external light sources, i.e., to control the external light variable. The main brick-walled room accommodated a large wooden room, which was, in turn, divided into two chambers: one housing the sample box and the other containing the measuring system. The brick-walled room was an air-conditioned, light-tight room, ensuring that both the system and samples (inside the wooden room) were maintained under strictly controlled light conditions. To further minimise the risk of any external photons entering through the camera lens, all the interior surfaces—including the inner walls of both chambers and the sample box—were covered with black plastic. Additionally, to prevent any potential light leakage or emissions from the walls and other structural elements, the exterior surfaces of the wooden room (containing both chambers) were lined with thick black fabric.

### 2.3. Measurement Procedure

Because the CCD camera is extremely sensitive, it was crucial to establish the initial conditions in complete darkness for accurate system calibration. The equipment was pre-calibrated and pre-aligned in situ by the manufacturer’s technical team using light standards of known and controlled intensity. Moreover, such calibration and validation procedures were routinely performed during their scheduled maintenance visits. To further ensure the measurement accuracy, two types of background acquisitions were performed in total darkness inside the nested dark rooms. The first acquisition, intended to establish a total zero level—comparable to an “absolute black”—was obtained by closing the camera shutter, thus blocking all optical elements going through the measurement path. The second acquisition recorded the background emission under standard measurement conditions (with the shutter open and the sampler in place but without any sample) and was performed, at least, immediately before and after each sample measurement session to effectively subtract ambient noise and interference. These blanks were captured under the same room conditions and experimental parameters as those used for the fruits’ measurements.

Furthermore, all the experimental parameters were optimised to minimise the noise and maximise the image resolution. The CCD camera was maintained at −100 °C via an external cooling system to reduce the thermal noise, while the other parameters such as acquisition mode (single scan), acquisition time (600 s), grating groove density (299.982 lines/mm), grating blaze (500 nm), input side slit (2500 μm), exposure time (600 s), pixel readout rate (5 MHz; this readout rate was the slowest possible to introduce the least noise), vertical shift speed (11.29 μs), pre-amplifier gain (2×) and horizontal and vertical binning (16 × 16) were selected based on previous optimisation studies [36,37]. Hence, these meticulous calibration and background correction procedures ensured that our UPE measurements were both accurate and reproducible, with minimal interference from environmental factors.

The UPE images were acquired between 10 AM and 2 PM for all fruits to minimise the influence of external variables. Prior to the measurement, the fruits were kept in complete darkness for 120 min to eliminate the residual effects of any previous light exposure, thereby avoiding DLE influenced by their surroundings, which is known to persist for some time after the light exposure. The fruits were organised into three sample sets as described in Section 2.1.

On the one hand, for the temporal evolution study, preliminary tests were conducted using various exposure times ranging from 1 to 30 min. These tests revealed that exposures longer than 10 min led to excessively high UPE in some fruits, resulting in sensor saturation due to the strong absorption of light. However, exposures shorter than 10 min did not sufficiently stimulate the induced UPE for any reliable analysis. Consequently, a 10 min exposure to natural sunlight was selected to provide a stimulation that allowed for the accurate evaluation of induced UPE while facilitating the subsequent observation of spontaneous UPE stabilisation. Following this 10 min exposure, the six fruits of each set were immediately transferred to the measurement room, arranged in the custom sampler, and measured at 10 min intervals over a total period of 180 min.

On the other hand, for the light exposure analysis, each sample set was placed in the big dark room, inside the designed box. The fruits were then frontally exposed for 10 min—consistent with the exposure time used in the temporal evolution study—to each artificial coloured light, using an RGB stand lamp (Lexman ADEO, France or to natural sunlight (by opening the blinds of an adjacent room’s window). Immediately after light exposure, the samples were transferred to the small dark room, where they were measured to capture induced UPE. In a separate experiment, following the same light exposure protocol, the samples were allowed to remain in complete darkness for 60 min to evaluate spontaneous UPE, after which UPE images were recorded for 10 min.

After these procedures, the experiments were repeated to obtain another replicate for all the measurements of both studies (temporal evolution and light exposure). Furthermore, throughout these experiments, the environmental conditions were rigorously controlled to minimise the influence of any potential confounding variables, such as temperature and humidity, which are known to affect the UPE measurements. Although the environmental conditions of our geographical region and the season during which the experiments were conducted (winter) ensured minimal ambient variability, we explicitly monitored these parameters to guarantee accurate and reproducible results.

The temperature within the measurement room was maintained at 15 °C via an air conditioning system and continuously monitored using an in-room thermometer. The temperature measurements were recorded both before and immediately after the light exposure, revealing a maximum variation of only approximately 0.5 °C, thereby confirming the stability of the experimental conditions.

The humidity levels were also tracked using a calibrated hygrometer, with no significant fluctuations observed during the experiments. This meticulous control of the environmental variables ensures that the observed UPE responses are solely attributable to the experimental protocols rather than the external climatic influences.

### 2.4. Data Analysis

The raw images were initially visualised using the Andor Solis 64-bit software v. 4.32.30065.0 (Oxford Instruments, Belfast, UK). Due to the cosmic ray interference, many pixels in these raw images exhibited abnormally high-intensity values. To address this, the raw images were processed using a custom algorithm developed in MATLAB R2024b (The MathWorks, Natick, MA, USA), which represents an updated version of the algorithm employed in our previous UPE studies [36,37]. This updated version retains the same core functionality while modifying the design of the region of interest (ROI) to adapt it to the various samples measured in each study, in this case, fruits.

The processing workflow of the algorithm is as follows: The algorithm begins by importing the original images and cropping them to a standard size to ensure uniformity across all samples. Subsequently, a 3 × 3 median filter is applied to effectively remove cosmic ray artefacts, thereby eliminating abnormally saturated pixels. Following this, all images—including both sample images and their corresponding background images—are normalised and denoised to ensure direct comparability. Thereafter, background subtraction is performed to remove environmental influences and further minimise noise and interference.

To extract quantitative data, an ROI is manually delineated for each fruit by creating a specific mask that exclusively includes the fruit. This step was implemented to deliberately exclude non-fruit regions, thereby enhancing the accuracy of the quantitative determination of UPE for each fruit. Similarly, this procedure ensured that the final UPE intensity measurement was exclusively attributable to the emission of the selected fruit. Once the ROI is defined, the algorithm calculates the average UPE intensity and standard deviation for the pixels within the ROI. The final outputs of the algorithm include (a) a corrected image with the selected ROI; (b) a 3D visualisation of the fruit’s intensity surface, where colours represent varying intensity levels (with red indicating maximum intensity and blue indicating minimum intensity); and (c) a histogram displaying the cumulative frequency distribution of intensity values within the ROI. By summing the frequencies from this histogram, a single numerical value is derived that quantitatively represents the UPE intensity of the selected area. This total sum represents the average UPE emission for each fruit, effectively transforming originally qualitative elements (the images) into quantitative elements (UPE intensity in numerical form), thereby allowing for an objective comparison of all the acquired data. A flowchart illustrating the operation procedure is provided in the Supplementary Material (Figure S1).

## 3. Results and Discussion

### 3.1. Temporal Evolution of Fruits’ UPE

The temporal evolution of UPE in fruits represents a central aspect of the study of biophoton emission from living systems, particularly due to the limited research led in this area and the need for an appropriate dark adaptation period to eliminate DLE (induced UPE) and isolate spontaneous UPE. While UPE has been more extensively studied in other biological systems, such as plants and humans, where it has been established that 20–30 min in darkness is sufficient for UPE to stabilise from its induced state to a spontaneous state, the same should not be assumed for fruits.

Although fruits originate from plants and serve a crucial biological function in supporting seed development, they are not considered living organisms in the traditional sense. Therefore, despite sharing some cellular characteristics with plants, the oxidative metabolism and chemical composition of fruits might influence their UPE behaviour differently. Thus, a deep examination of the temporal dynamics of fruits’ UPE is necessary to establish the required stabilisation period and to discern whether this time varies among different types of fruits or is influenced by their external colour.

As a consequence, to accurately measure the evolution of fruits’ UPE over time, the designed protocol was meticulously followed (see Section 2.3). The short-duration measurements (10 min) enabled us to comprehensively evaluate the impact of light on UPE in various fruit types over an extended and helped to differentiate between induced and spontaneous UPE in the different samples.

**Figure 2:**
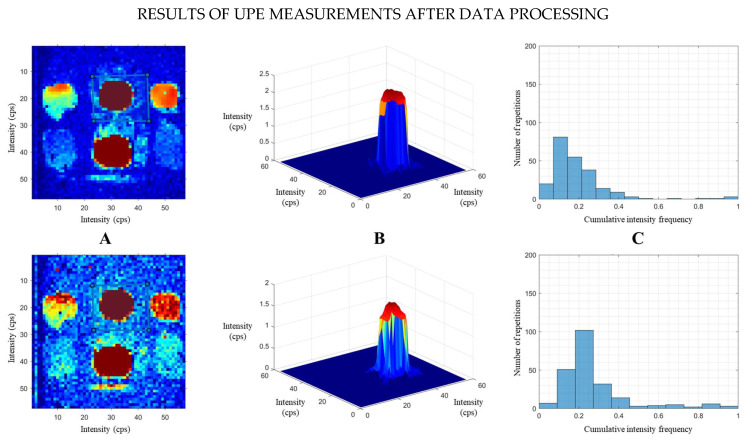
Example of the UPE measurements after data processing, showing the corrected images (**A**), 3D surface images (**B**) and histograms (**C**) of two consecutive measurements of Set A.

Thus, Figure 2 illustrates an example of the results, showing the corrected images of two consecutive measurements of Set A. The silhouettes of the fruits are clearly observed within the selected ROI for each sample. The horizontal sequences display the corrected images of the fruits with their custom ROI (A), the 3D surface visualizations (B) and the histograms (C).

The ROI shown in the masked image for each fruit was created by applying a manually drawn mask to the original image. In other words, six distinct masks were created for each set of fruits (with four masks designed for citrus fruits). The homemade algorithm enabled us to analyse the images of all fruits simultaneously by selecting a different mask for each fruit and processing the fruit within it.

**Figure 3:**
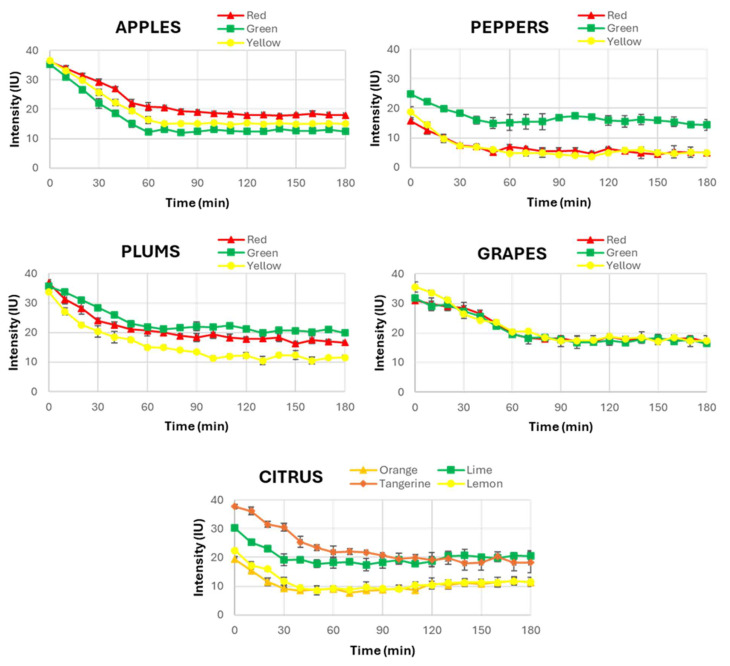
Temporal evolution of UPE in the three selected sets of fruits, including the average values of UPE intensity and their standard deviations in intensity units (IU).

As a result, Figure 3 provides a complete view of the temporal evolution of UPE across the different fruits (apples, peppers, plums, grapes and citrus fruits). A consistent pattern was observed in most of them, where UPE exhibited a decline within the first 60 min after light exposure. This decrease in UPE intensity values indicates the dissipation of the induced UPE, approaching a stabilised state indicative of spontaneous UPE. This finding is fundamental as it challenges the assumption that all biological systems have the same behaviour in terms of UPE stabilisation, a fact that has not been investigated in other research involving UPE in fruits.

Then, this observation suggests that while plants generally behave similarly to humans in their UPE response, fruits exhibit prolonged periods of UPE stabilisation. The slower stabilisation in fruits could be attributed to differences in their unique metabolic activities, such as variations in their oxidative metabolism or the structural characteristics of their skin and flesh. These differences may influence how they absorb and re-emit light, especially when compared to plants and humans.

Interestingly, despite examining fruits of various external colours, no significant correlation was found between the colour of the fruit and the time required for UPE stabilisation. This finding indicates that while the external colour might influence the intensity of UPE, it does not seem to affect the temporal dynamics of its stabilisation. Therefore, the colour of the fruit should not be considered a determining factor in the duration needed to reach spontaneous UPE. This result simplifies the experimental procedure for future research, allowing a focus on the fruit type rather than its colour when determining the necessary dark adaptation period prior to UPE measurements. Moreover, this fact demonstrates that among all the factors that can influence UPE in the stabilisation time to be completely spontaneous, the pigmentation of the fruits is not one of the most relevant.

Based on these findings, the same UPE stabilization period was selected for all the studied fruits, and it was concluded that 60 min of dark adaptation ensures the transition from induced to spontaneous UPE for all fruits. The UPE intensity curves consistently show a decline, and, after 60 min, the stabilization intensity level has been reached in all the fruit types studied, confirming that this duration was required to eliminate any residual emission from prior light exposure. Therefore, it was decided to standardise a dark adaptation period of 60 min for the measurement of spontaneous UPE in the second part of this study, where the influence of external light on fruits’ UPE was examined. This ensured that only the effect of the selected light in each case was evaluated, thus avoiding the possible influence of prior environmental light.

The lack of specific studies on the temporal evolution of UPE in fruits underscores the novelty and importance of our findings. Although variations in stabilisation time could theoretically occur depending on fruit type or environmental conditions, our results demonstrate remarkable consistency across different species, suggesting that this 60 min period is broadly applicable. By requiring a dark adaptation period of 60 min, we provide a new benchmark for future UPE studies with fruits. This ensures that true spontaneous UPE is measured without the confounding effects of residual induced emission due to environmental light exposure.

### 3.2. Influence of External Light on Fruits’ UPE

To investigate the influence of external light on the fruits’ UPE, samples were exposed for 10 min to various light sources (both artificial and natural). Immediately after light exposure, induced UPE was measured. In a separate protocol, samples remained in complete darkness for 60 min to enable the stabilisation of spontaneous UPE before measurement. All measurements were carried out using the custom-designed sampler within the respective sample sets (see Section 2.1 and Section 2.2).

**Figure 4:**
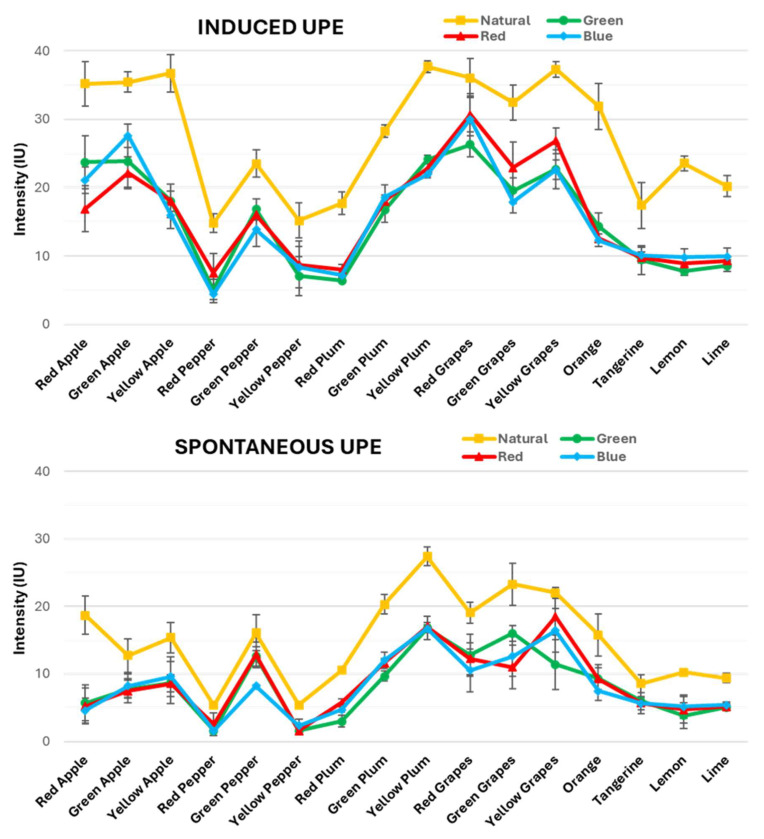
Induced UPE (top) and spontaneous UPE (bottom) intensity values of the studied fruits, showing the effects of the four different lights used. Fruits are ordered according to the selected sets of samples. The graphs also report the average values of the UPE intensity and their standard deviations in intensity units (IU).

Consequently, Figure 4 illustrates the results of the induced and spontaneous UPE measurements from the chosen fruits, organised into two distinct graphics, respectively. The four different lines in the graphs correspond to the selected light sources: red, green and blue lights and sunlight. These fruits are ordered in the X-axis according to their types and colours, as clustered in the mentioned sets of samples. This arrangement allowed us to clearly see how each type of light impacts the UPE of these fruits, which were further categorised into their respective sets to facilitate comparison.

Furthermore, additional visuals are provided in the Supplementary Material (Figures S2–S6), where there are alternative graphics displaying the induced and spontaneous UPE results for all the fruits individually. These graphics allow for a closer analysis of the UPE intensity values for each single fruit under the four studied light sources. With this supplementary visualization of the data, a more comprehensive study of the results can be performed, leading to the reveal of similarities and differences that might be less evident in the primary graphics presented in the main text.

First, comparing both graphs, it is observed that induced and spontaneous UPE follow the same trend. However, the intensity of the induced UPE is consistently higher than that of the spontaneous UPE across all light conditions and fruit types. This pattern remained constant regardless of the fruit’s variety or colour, which is explained by the fact that the induced UPE is due to prior light exposure. However, the spontaneous UPE arises solely from the natural emission of cells.

Analysing the first graph (Figure 4, top) reveals significant variations in the induced UPE values across different light exposures among the fruit types. Under natural light, the induced UPE is consistently elevated compared to the artificial lights, showing a range of intensities from 15 to 35 IU. Notably, it can be observed that certain fruits, such as apples and grapes, reached around 35 IU across their three colour variations with minimal variability. However, peppers and citrus fruits displayed values at around 20 IU, with greater variability among their colour variations. Likewise, plums ranged between 20 and 35 IU, showing substantial variability across the three colour variations.

This suggests that natural light maximally activates UPE in all fruits. This effect could be due to the fact that natural sunlight is a full-spectrum light source covering a wide range of wavelengths, including the entire VIS light range (400–800 nm). For that reason, it effectively stimulates more the fruit tissues compared to the artificial lights that emit only a narrow wavelength range. It is important to remember that the UPE light range is maximally produced in the VIS range, and then the broad spectral composition of the sunlight leads to higher photon absorption by the fruit’s surface, thereby generating a stronger induced UPE response. This is reflected by the higher UPE values observed under natural light conditions compared to those obtained with artificial RGB lights.

Among the artificial lights, the induced UPE values were generally lower than for natural light, with intensities ranging from 5 to 30 IU. Hence, when examining the induced UPE responses by fruit type under artificial light, apples and grapes again exhibited the highest values (~25 IU). In turn, the variable values of plums (10–25 IU) were lower, while the results of peppers and citrus fruits (~10 IU), were the lowest but more consistent than the values observed for plums. This trend indicates that while artificial light can elicit an increase in induced UPE, its effects are limited compared to natural light. Moreover, there is minimal distinction among the colours of the artificial lights. These findings highlighted that the induced UPE emission was influenced by the light source. As expected, natural light was the most effective compared to any of the artificial lights used.

In Figure 4 (bottom), the spontaneous UPE values are shown after a 60 min dark adaptation period, which stabilises the UPE baseline for all fruit types under each light exposure. Consistently, the spontaneous UPE values are lower than the induced UPE values across all fruit types, colours and lights, ranging between 1 and 25 IU. Under natural light, the spontaneous UPE values are slightly elevated compared to the values for artificial lights. However, this increase is subtle and not as pronounced as in the induced UPE values. For instance, apples and grapes presented the highest spontaneous UPE values, reaching around 15 and 20 IU, respectively, and with little variability among their colour variations. However, plums showed again considerable variability, ranging from 10 to 25 IU, while peppers and citrus fruits did not exhibit as much variability, with values around 10 IU.

Across all artificial lights, the spontaneous UPE values generally stabilised at lower intensities than under natural light, ranging from 1 to 20 IU. In particular, grapes showed the highest values for these three lights, reaching between 10 and 20 IU. Likewise, plums followed with their typically variable intensities ranging from 5 to 15 IU, whilst peppers continued this trend with values ranging from 1 to 10 IU. Finally, apples and citrus fruits showed the lowest values among all these fruits, ranging between 5 and 10 IU, although the variation among their colour types was less pronounced compared to the other fruits. As we can observe, even if these values varied, the differences among the different lights were not as marked as in the induced UPE values, confirming that the spontaneous UPE baseline had a similar pattern regardless of the previous exposure to any light source.

Thus, this study underscores the differential impact of light sources on the fruits’ UPE and the importance of considering both the induced and spontaneous UPE when analysing the effects of light.

### 3.3. Differentiation of Organic and Conventional Fruits by UPE

The distinction between organic and conventional fruits has traditionally been challenging to establish through chemical analysis, as previous studies have often failed to identify significant differences using common analytical techniques. Thus, the current study aimed to investigate whether spontaneous UPE, produced by the biochemical properties of fruits, could offer a novel approach to this differentiation. This analysis is particularly relevant given the increasing interest in forensic science to address food fraud, specifically in cases where non-organic fruits may be mislabelled and sold as organic, yielding illicit financial benefits due to the higher market value of organic products.

For this exploratory study, a small but representative sample was selected, consisting of a red apple, a red pepper and a green pepper—all certified organic. These fruits were chosen to provide a variety of species and colours while also representing common fruits that are available in both organic and conventional forms. The decision to focus on a limited sample was dictated by the practical challenges associated with sourcing organic fruits. This is because not all fruit varieties are commercially available in their organic form, and those that are can be difficult to find in conventional retail environments.

**Figure 5:**
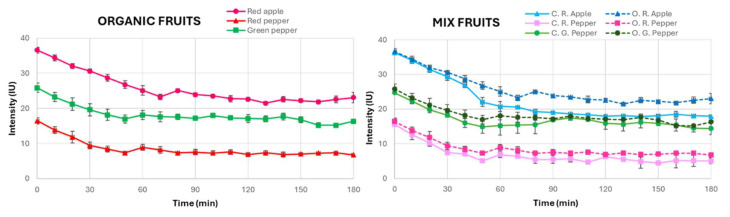
Temporal evolution of UPE in organic fruits (left) and both organic and conventional fruits (right). The graphs show the average values of UPE intensity and their standard deviations in intensity units (IUs).

The experimental procedure was the same as the one used for conventional fruits, with the organic fruits subjected to 120 min of complete darkness, followed by 10 min of exposure to natural sunlight, and the subsequent UPE measurements every 10 min for a total period of 180 min. Figure 5 shows the temporal evolution of the UPE measurements from organic fruits.

The results of this analysis showed that, like conventional fruits, organic fruits also required 60 min to stabilise their UPE. This indicates that both organic and conventional fruits exhibit comparable biophotonic behaviours in terms of stabilisation kinetics, suggesting that the dark adaptation time alone is not a sufficient metric for differentiation. However, a notable observation was that the organic fruits consistently exhibited slightly higher UPE intensities than the conventional ones across all three types of fruit studied. Although this difference was modest for red and green peppers, it was more pronounced in apples, implying that the distinct biochemical properties of each fruit type may lead to a differential UPE response. In addition, this effect of the biochemical processes on the UPE measured for organic vs. conventional crops seems to be independent of the fruit’s ripeness stage, as deduced from the similar variation between green and red peppers.

These differences in UPE intensity may reflect underlying variations in metabolic processes of organic produce, particularly in the production of ROS, which are vital to UPE. While alternative explanations for these observations have been considered, we regard the increased environmental stress associated with organic cultivation (e.g., limited synthetic inputs or higher biotic and abiotic pressures)—resulting in enhanced ROS production as part of plants’ adaptive responses—as the most plausible explanation. This interpretation is supported by previous studies in other biological systems; for instance, research on berries and eggs has reported higher UPE emissions in organic samples, attributed to enhanced oxidative metabolism and stress-induced ROS activity [38,39,40]. In apples, where the divergence in intensity was most marked, the higher UPE could be linked to their unique biochemical composition and a greater propensity for stress-induced ROS generation compared to peppers. These findings suggest that UPE measurements may serve as a sensitive biomarker for subtle metabolic differences influenced by cultivation practices, thereby providing a novel tool for forensic differentiation between organic and conventional produce and helping in food fraud detection.

## 4. Conclusions

This study offers a comprehensive analysis of UPE in various fruit types under different lighting conditions, significantly contributing to the understanding of how spontaneous and induced UPE behave when exposed to different stimuli over time. From a forensic perspective, the ability to measure and differentiate UPE across various fruits and under distinct lighting conditions could provide a non-invasive and novel method for detecting biological or environmental changes that is potentially valuable in forensic investigations where biological materials are involved.

The data demonstrated that the induced UPE was consistently higher than the spontaneous UPE across all light sources, fruit types and colours. The induced UPE displayed greater variability among the different light sources, particularly under natural light, while the spontaneous UPE showed more stability. This latter result confirmed that the spontaneous UPE remained largely unaffected by prior light exposure. In turn, this reinforced its potential reliability as a forensic tool for analysing biological samples, regardless of the lighting conditions to which they have been exposed. The methodology used, establishing a 60 min dark adaptation period due to the results of the temporal evolution, ensured that the spontaneous UPE measurements were accurate, providing a solid baseline that can be used in forensic contexts to assess the natural biophoton emissions of a biological sample without external influence. This is a critical finding that has not been investigated before.

Moreover, the differences between fruit types were particularly notable. Apples, plums and grapes exhibited significantly higher UPE levels, both spontaneous and induced, compared to peppers and citrus fruits. This finding suggests that UPE is closely linked to the biological characteristics of the sample, such as cellular composition or biochemical properties, which could provide a valuable forensic marker for differentiating between biological materials. The slight variations within fruit types further indicated the potential for UPE analysis to detect subtle differences within the same category, potentially offering a tool for identifying specific biological traits or conditions.

Overall, the findings of this research highlighted the clear effect of light type on the UPE, with natural light eliciting the strongest UPE response. Therefore, one of the key findings was that natural light stimulated higher UPE levels—both spontaneous and induced—compared to artificial coloured lights. This result aligns with expectations, given that sunlight encompasses the full electromagnetic spectrum, while coloured artificial lights are limited to a single or very narrow wavelength range.

This study also underscores the importance of considering both the type of biological material (e.g., different fruits) and the lighting conditions when analysing UPE, as these factors significantly influenced the results. From a forensic analysis perspective, these insights are particularly valuable as they suggest that the UPE measurements could serve as a non-invasive method for assessing biological samples in forensic contexts, potentially aiding in various criminal studies, such as the evaluation of environmental conditions at crime scenes or, especially, the investigation of food frauds.

Moreover, the additional analysis of UPE in organic fruits suggested that while the temporal stabilisation of UPE is similar between organic and conventional fruits, there may be differences in UPE intensity that require further investigation. This preliminary finding opens the possibility of using UPE as a novel tool for distinguishing between organic and conventional fruits, offering a potential new approach in the forensic examination of the origin of the food.

However, in real-world scenarios, applying UPE measurements for differentiating organic vs. conventional fruits faces several challenges. These include the need for controlled measurement conditions (e.g., maintaining strict darkness, constant temperature and humidity), variability among the fruit samples and the requirement for specialised, small yet highly sensitive instrumentation. Additionally, the environmental factors during the sample collection and handling could affect UPE readings, necessitating rigorous standardisation protocols to ensure reproducibility and reliability in forensic contexts. We are aware that current technological limitations hinder the practical deployment of such measurements in the field. Nevertheless, we anticipate that as technology advances—particularly with the instrumentation miniaturisation—these challenges will be progressively overcome, thereby facilitating the robust application of the UPE measurements in real-world scenarios while maintaining all the required controlled conditions for this low-intensity emission.

As a general conclusion, this study represents a significant step towards understanding the potential forensic applications of UPE in biological materials, particularly in fruits. The clear distinctions observed between UPE levels in different fruit types, under varying light conditions, open the door to the possibility of using UPE as a tool in forensic science. Future research could expand on these findings by investigating UPE emissions in other biological materials and under different environmental conditions, further establishing the role of UPE as a valuable forensic marker. Thus, the results of this study contribute to advancing the use of UPE in forensic science, offering a new perspective on the analysis of biological samples through a non-invasive, light-based method.

## Data Availability

The raw data supporting the conclusions of this article will be made available by the authors on request.

## Supplementary Materials

The following supporting information can be downloaded at: https://www.mdpi.com/article/10.3390/s25061799/s1, Figure S1: Algorithm functioning flowchart. Figure S2: Histograms of induced UPE (left) and spontaneous UPE (right) intensity values of apples, illustrating the effects of the four different lights used. The graphs show the average values and their standard deviations in intensity units (IU). Figure. S3: Histograms of induced UPE (left) and spontaneous UPE (right) intensity values of peppers, illustrating the effects of the four different lights used. The graphs show the average values and their standard deviations in intensity units (IU). Figure S4: Histograms of induced UPE (left) and spontaneous UPE (right) intensity values of plums, illustrating the effects of the four different lights used. The graphs show the average values and their standard deviations in intensity units (IU). Figure S5: Histograms of induced UPE (left) and spontaneous UPE (right) intensity values of grapes, illustrating the effects of the four different lights used. The graphs show the average values and their standard deviations in intensity units (IU). Figure S6: Histograms of induced UPE (left) and spontaneous UPE (right) intensity values of citrus fruits, illustrating the effects of the four different lights used. The graphs show the average values and their standard deviations in intensity units (IU).
