# Supplementary material for: Influence of External Light on Ultra-Weak Photon Emission of Fruits: Forensic Differentiation of Organic and Conventional Fruits
Source: Sensors (Basel). 2025 Mar 14;25(6):1799. doi: 10.3390/s25061799 (PMC11946304; doi:10.3390/s25061799)
Supplement: Supplementary file 1 [file sensors-25-01799-s001.zip › sensors-3502721-supplementary.pdf]

Influence of external light on ultra-weak photon emission of fruits: Forensic differentiation of organic and conventional fruits

Supplementary Material

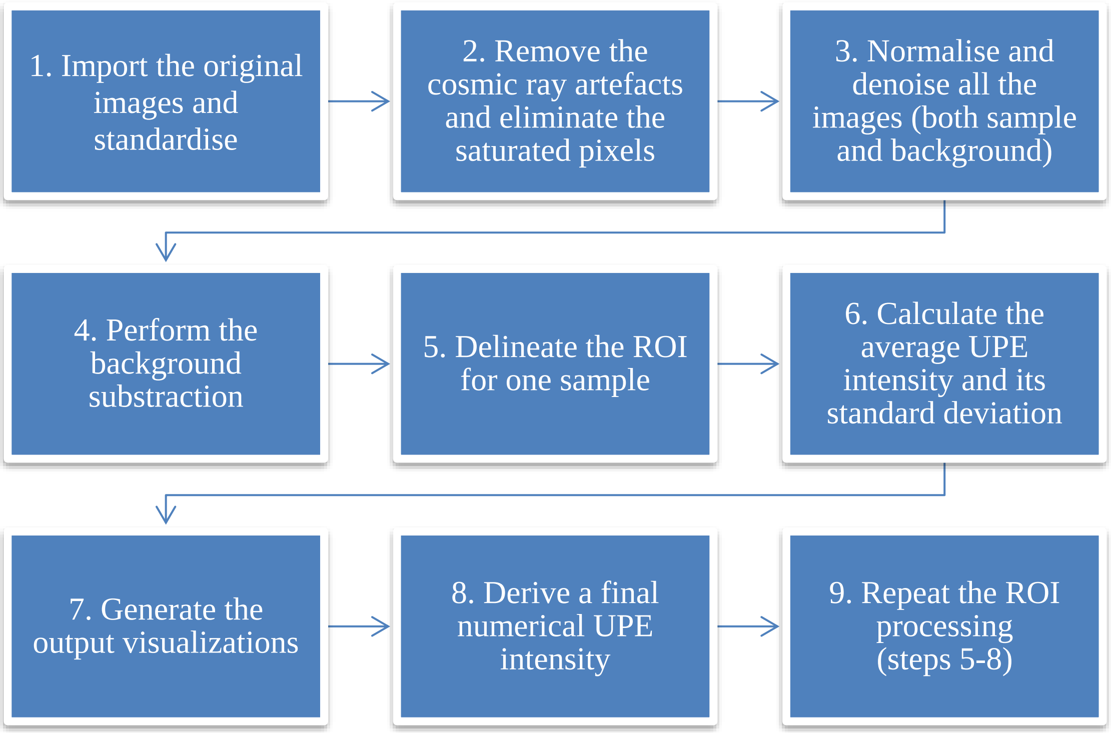

Fig. S1. *Algorithm functioning flowchart.*

RESULTS OF UPE MEASUREMENTS: *APPLES*

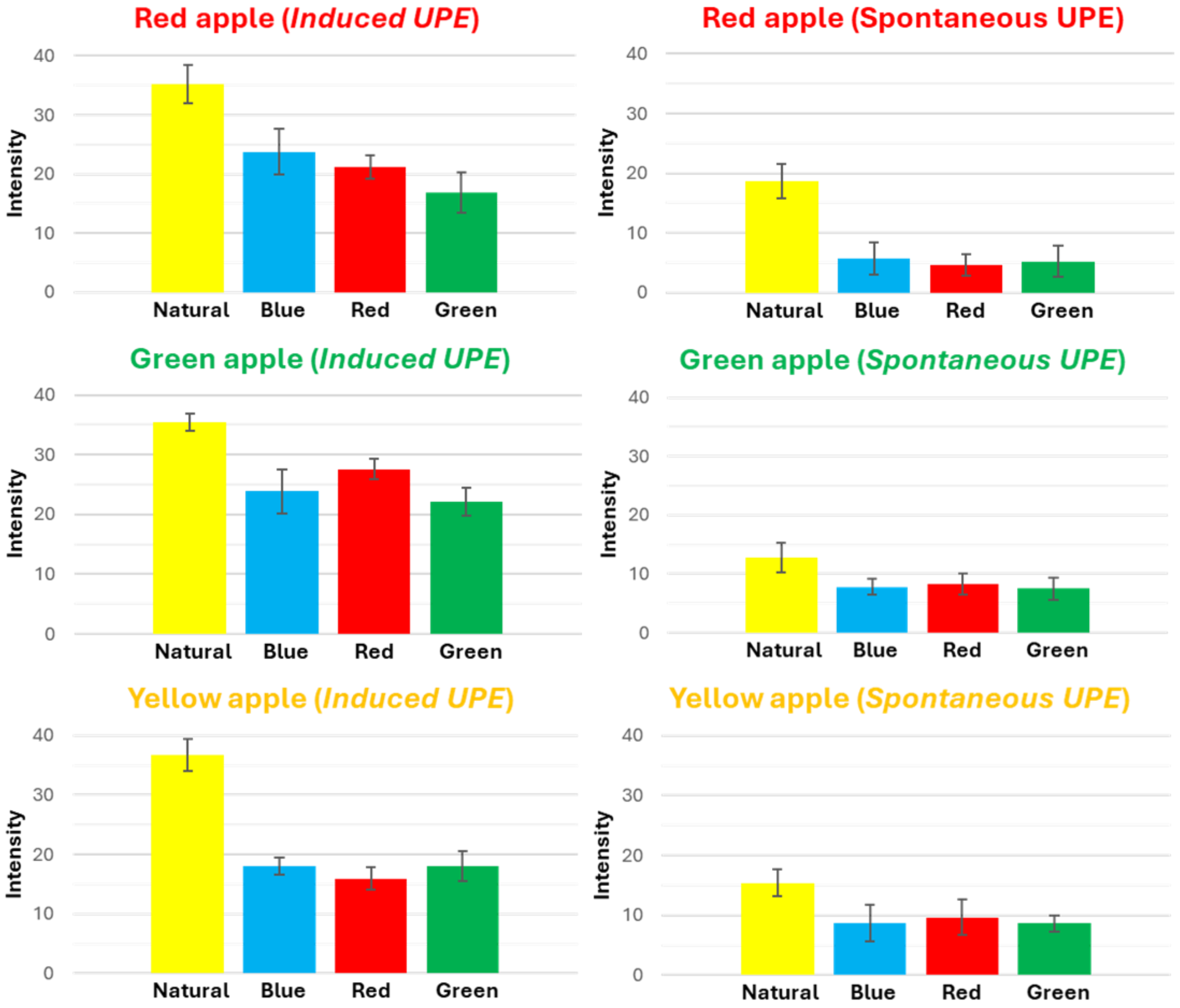

Fig. S2. *Histograms of induced UPE (left) and spontaneous UPE (right) intensity values of apples, illustrating the effects of the four different lights used. The graphs show the average values and their standard deviations in intensity units (IU).*

RESULTS OF UPE MEASUREMENTS: *PEPPERS*

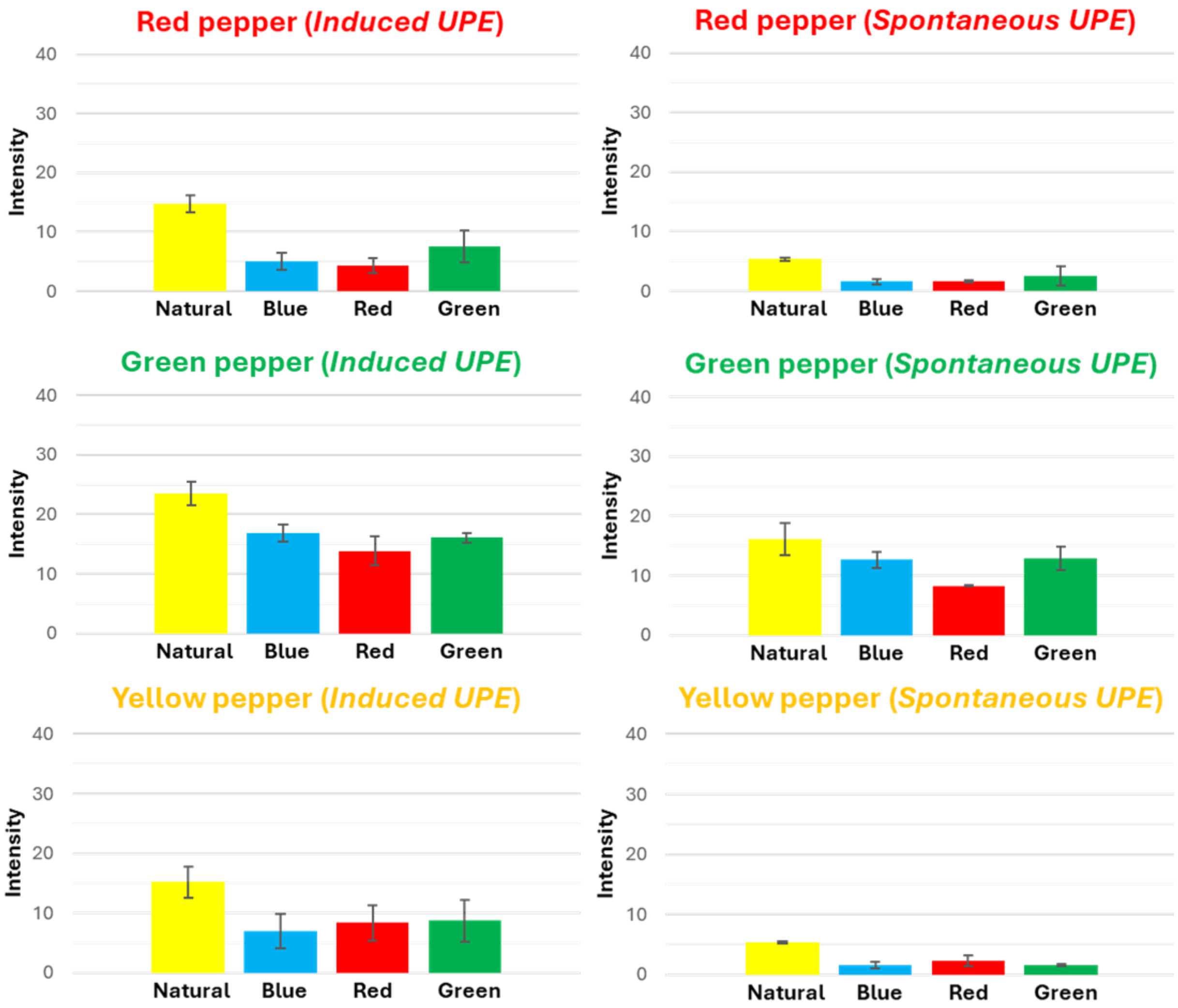

Fig. S3. *Histograms of induced UPE (left) and spontaneous UPE (right) intensity values of peppers, illustrating the effects of the four different lights used. The graphs show the average values and their standard deviations in intensity units (IU).*

RESULTS OF UPE MEASUREMENTS: *PLUMS*

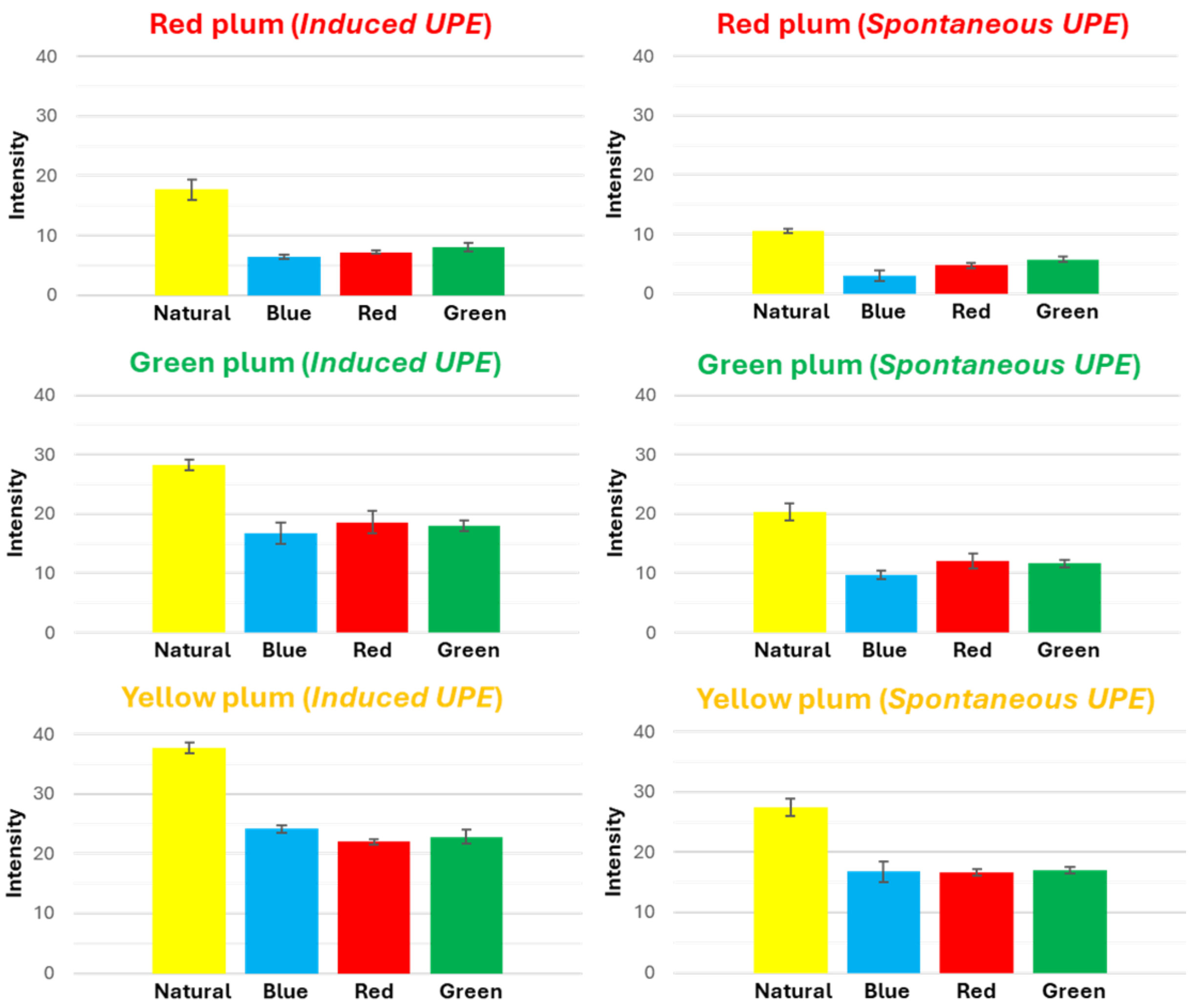

Fig. S4. *Histograms of induced UPE (left) and spontaneous UPE (right) intensity values of plums, illustrating the effects of the four different lights used. The graphs show the average values (intensity units, IU) and their standard deviations.*

<u>RESULTS OF UPE MEASUREMENTS: *GRAPES*</u>

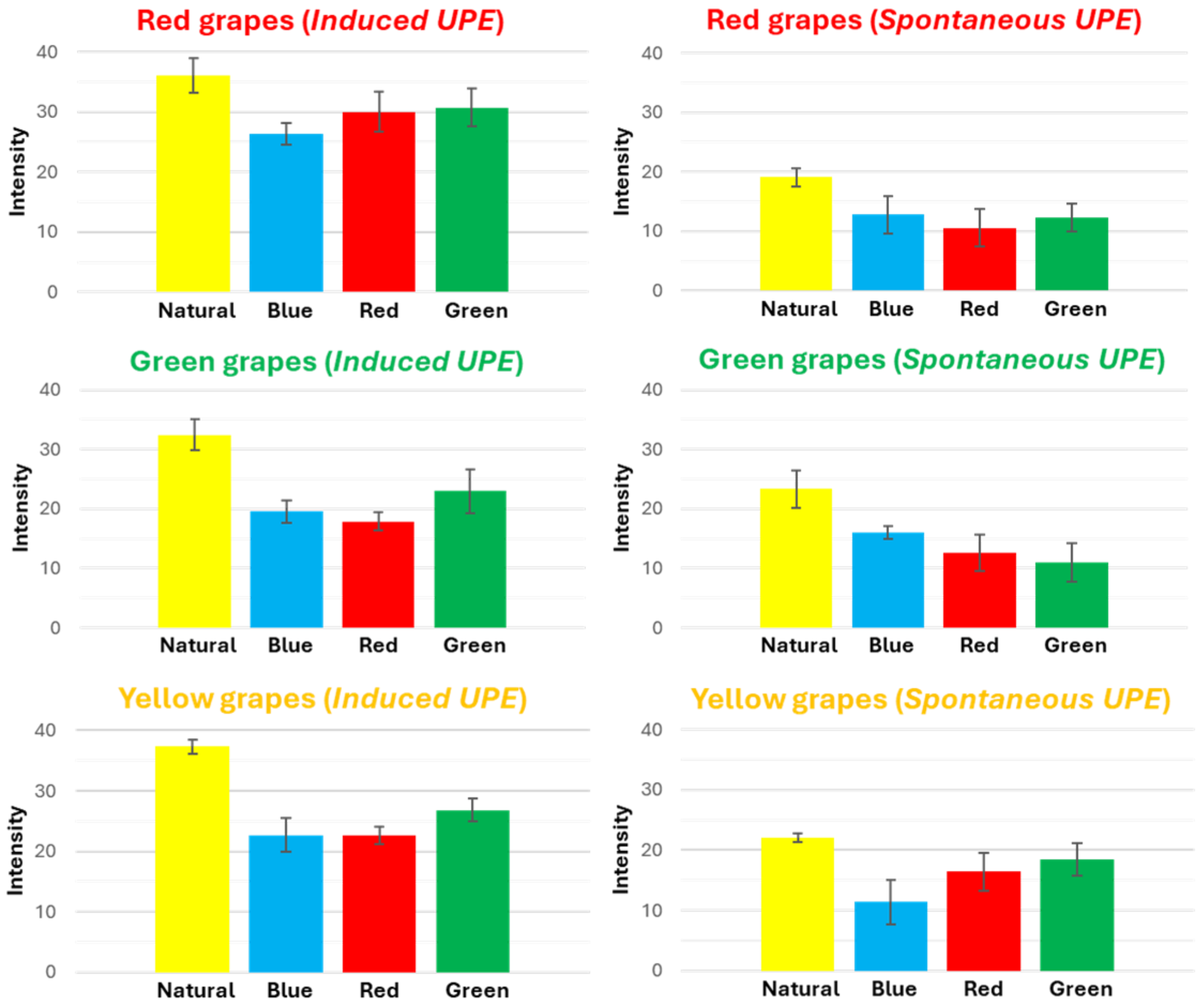

Fig. S5. *Histograms of induced UPE (left) and spontaneous UPE (right) intensity values of grapes, illustrating the effects of the four different lights used. The graphs show the average values and their standard deviations in intensity units (IU).*

<u>RESULTS OF UPE MEASUREMENTS: *CITRUS FRUITS*</u>

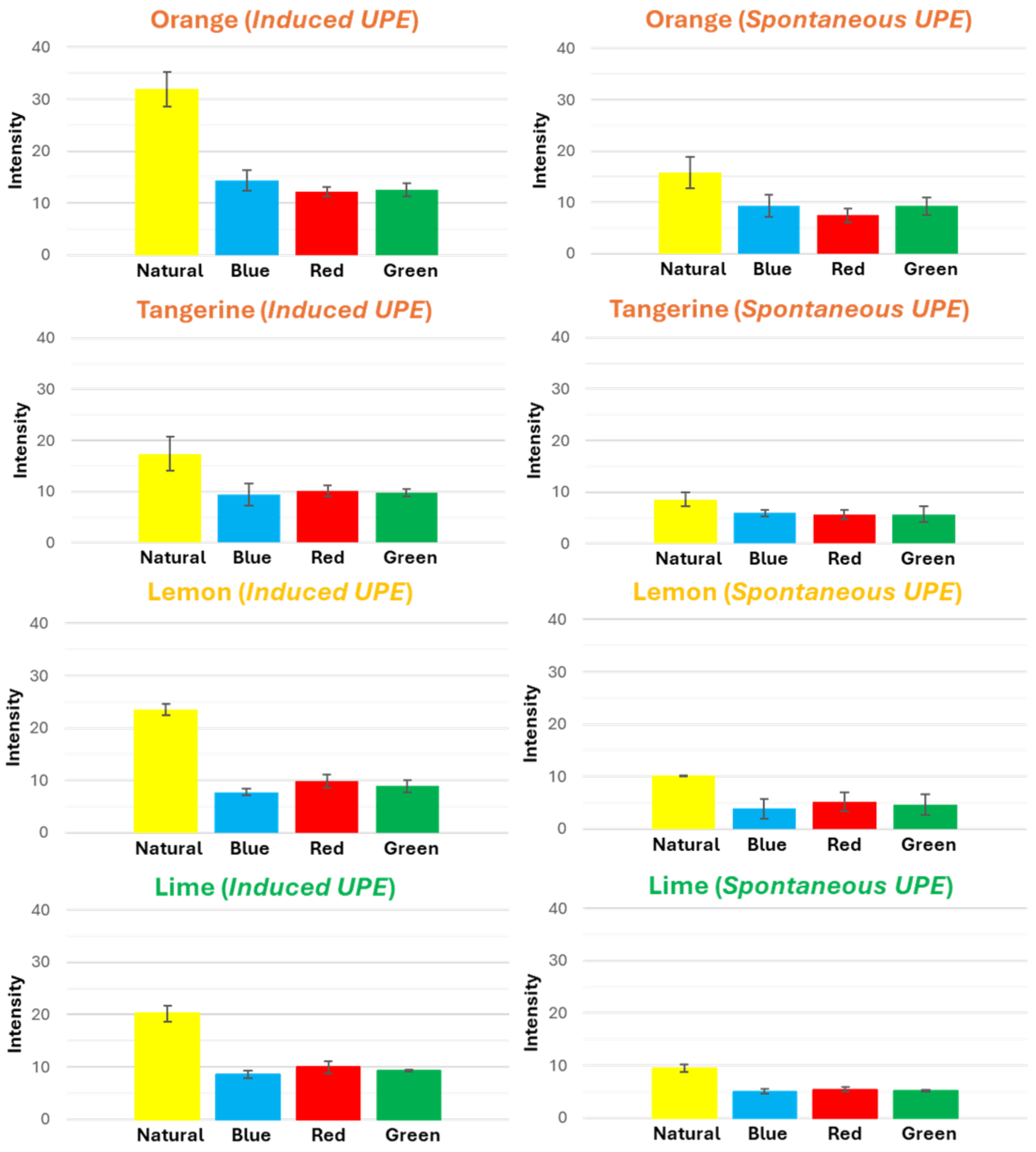

Fig. S6. *Histograms of induced UPE (left) and spontaneous UPE (right) intensity values of citrus fruits, illustrating the effects of the four different lights used. The graphs show the average values and their standard deviations in intensity units (IU).*
